# Carbamate-Based Bolaamphiphile as Low-Molecular-Weight Hydrogelators

**DOI:** 10.3390/gels2040025

**Published:** 2016-09-28

**Authors:** Laurent Latxague, Alexandra Gaubert, David Maleville, Julie Baillet, Michael A. Ramin, Philippe Barthélémy

**Affiliations:** ARNA laboratory, Univ. Bordeaux, ChemBioPharm, INSERM, U1212, CNRS UMR 5320, F-33000 Bordeaux, France; laurent.latxague@u-bordeaux.fr (L.L.); alexandra.gaubert@u-bordeaux.fr (A.G.); david.maleville@u-bordeaux.fr (D.M.); julie.baillet@outlook.fr (J.B.); michael.ramin@inserm.fr (M.A.R.)

**Keywords:** nucleolipid, nucleoside, glycosyl, bolaamphiphiles, low-molecular-weight gels (LMWG), carbamate, hydrogels, supramolecular assemblies, thixotropy

## Abstract

A new bolaamphiphile analog featuring carbamate moieties was synthesized in six steps starting from thymidine. The amphiphile structure exhibits nucleoside-sugar polar heads attached to a hydrophobic spacer via carbamate (urethane) functions. This molecular structure, which possesses additional H-bonding capabilities, induces the stabilization of low-molecular-weight gels (LMWGs) in water. The rheological studies revealed that the new bolaamphiphile **7** stabilizes thixotropic hydrogels with a high elastic modulus (*G*′ > 50 kPa).

## 1. Introduction

**Figure 1:**
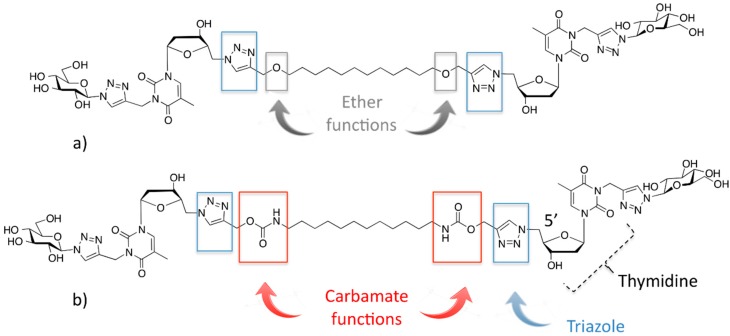
Schematic representations of bolaamphiphile structures. (**a**) Bolaamphiphile structure previously reported (see [15]). The hydrophobic segment is attached via ether functions. (**b**) Bolaamphiphile structures with carbamate functions described in this study.

Supramolecular gels [1] are of interest in biomedicine for several biomedical applications including biological assays [2], wound healing [3], tissue engineering [4], or drug delivery [5]. The glycosyl-nucleoside-lipids (GNLs) [6,7,8] family belongs to amphiphilic structures composed of biological units such as sugars, lipids, and nucleic acids. Because of their intrinsic supramolecular properties, these amphiphiles have been studied as low-molecular-weight gelators (LMWGs). Among GNLs, bolaamphiphiles [9,10,11,12,13,14], which are composed of a hydrophobic chain covalently linked at both ends to hydrophilic head groups, have been recently reported as biocompatible LMWG-based hydrogels suitable for the culture of stem cells [15]. The molecular architectures previously reported exhibit lipid moieties covalently attached via ether functions (Figure 1a). 

In order to improve the rheological properties of LMWG-based hydrogels, we modulated the bolaamphiphile architectures by inserting additional hydrogen bond functions. In this design, the hydrophobic segment is attached to the nucleobase via a triazole ring and a carbamate moiety at the 5′-position of thymidine.

Herein, we report the synthesis of a new carbamate-based bolaamphiphile, based on N-thymine glycosylated compounds as head groups and a lipid chain attached at the 5′-position of the nucleoside (Figure 1). The rheological studies revealed that such structures stabilize low-molecular-weight hydrogels at different concentrations including 1%, 3%, and 4%. 

## 2. Results and Discussion

### 2.1. Synthesis of the Carbamate-Based Bolaamphiphiles

In order to expend the current repertoire of the bolaamphiphiles, we selected the carbamate function to attach the hydrophobic central synthetic unit to the polar heads. The carbamate moiety, also called urethane, is a structural function used in many approved therapeutic compounds [16]. Its functionality, which is linked to amide-ester hybrid features, displays good chemical and biochemical stabilities. This function has been used in medicinal chemistry as a peptidic bond mimetic. Interestingly, the carbamate function can participate in hydrogen bonding through both the carboxyl and the NH motifs. Different hydrophilic and hydrophobic motifs can be connected to the carbamate O- or N-termini, or both. Hence, inserting this functionality into the GNL structures would allow us to modulate their supramolecular properties.

**Scheme 1:**
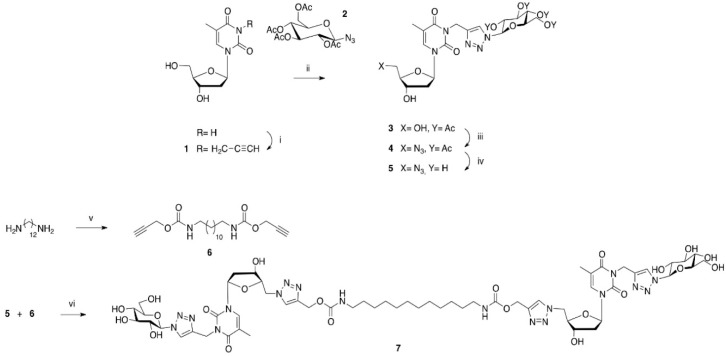
General synthesis (**i**) K_2_CO_3_, propargyl bromide, tetra-n-butylammonium iodide (TBAI), dimethylformamide (DMF), r.t., 12 h (81%). (**ii**) **2**, ^t^BuOH/H_2_O (1:1), CuSO_4_, ascorbic acid, 75 °C, 7 h. (**iii**) PPh_3_, NaN_3_, CBr_4_, DMF, r.t., 24 h. (**iv**) MeONa 1% in MeOH, r.t., 30 min. (**v**) Propargyl chloroformate, Et_3_N, CH_2_Cl_2_, r.t., 24 h. (**vi**) Tetrahydrofuran (THF)/H_2_O (1:1), CuSO_4_, ascorbic acid, 65 °C, 4 h.

The synthetic strategy developed to access to the new carbamate bolaamphiphile is based on a key intermediate **5**: the 5′-desoxy-*N*-3-[1-((β-d-glucopyranoside)-1*H*-1,2,3-triazol-4-yl)methyl] azidothymidine (compound **5**, Scheme 1). The commercial starting material (thymidine) was first alkylated at the position 3′ by using propargyl bromide in dimethylformamide (DMF) in the presence of potassium carbonate to give compound **1** (Scheme 1). The propargyl moiety was then “clicked” with a peracetylated azidoglucose to provide intermediate **3** with a 60% yield (Scheme 1). The peracetylated azidoglucose **5** was then prepared due to a substitution reaction (SN_2_) in the presence of triphenylphosphine (PPh_3_), sodium azide (NaN_3_), and tetrabromomethane (CBr_4_) in DMF. This reaction provided the protected azidoglycoside **4** (Scheme 1, III), which was finally subjected to Zemplen’s deacetylation to give the expected compound **5** (Scheme 1, IV). In parallel, the dicarbamate hydrophobic central synthetic unit **6** was prepared starting from commercial 1,12-diaminododecane. This diamine was reacted with the propargyl chloroformate to give the dicarbamate hydrophobic spacer **6** (Scheme 1, V). The latter was thus engaged in a click reaction with two equivalents of **5** to obtain the expected bolaamphiphile **7** with a 65% yield (Scheme 1, VI).

### 2.2. Physicochemical Studies

An important goal of the present study was to determine the impact of structural modifications (the insertion of carbamate) on the supramolecular properties. Thus, the physicochemical properties of compound **7** were studied, including (i) the stabilization of gel in water; (ii) the rheological properties; and (iii) the gel-sol transition temperature transition. 

#### 2.2.1. Gel Formation Assays

**Figure 2:**
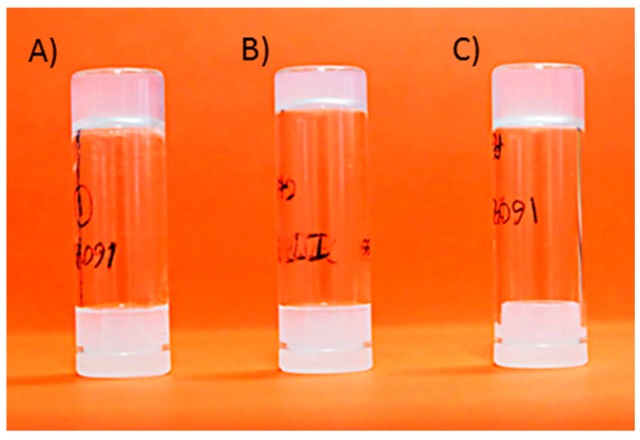
Hydrogel formation at 1% (**A**); 3% (**B**); and 4% (**C**) (*w*/*v*).

Bolaamphiphile carbamate **7** was dissolved in Milli-Q water to obtain gel concentrations of 1%, 3%, and 4% (*w*/*v*). To ensure complete dissolution of compounds, samples were heated at 60 °C and shaken at 1250 rpm for 30 min using a Thermomixer compact (Eppendorf, Hauppauge, NY, USA). Samples were cooled down at 4 °C for one day (gel at 3% and 4% (*w*/*v*)) or two days (gel at 1% (*w*/*v*)). The samples were allowed to return at room temperature. In order to evaluate the gel formation, samples were then turned upside-down; no flowing under its own weight indicated sample gelification. As shown in Figure 2, compound **7** enabled homogeneous hydrogel formation at low concentrations. 

Gel formation was also assessed at 25 °C. However, using the same parameters (sonication, heating, and storage at RT), no homogeneous gel was observed. After incubation at 25 °C, gel formation occurred at the bottom of the test tube, and some non-gelified water remained above. Longer storage at room temperature (2 or 3 days up to one week) did not improve the stabilzation of the sample.

#### 2.2.2. Rheological Studies

**Figure 3:**
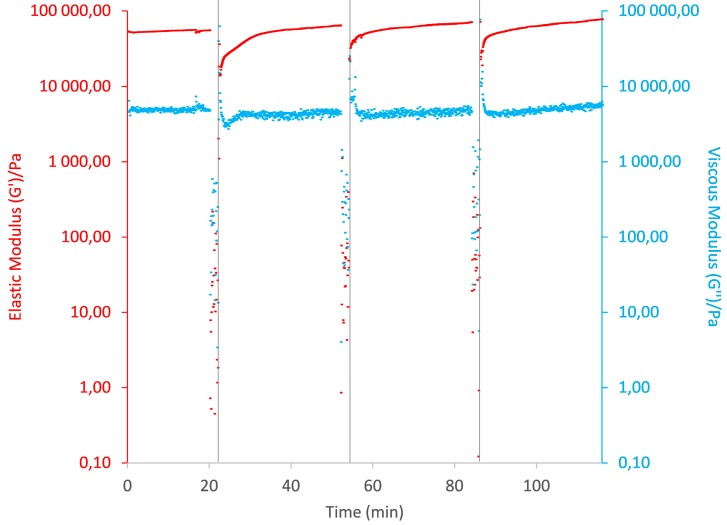
Step-strain measurement of 4% (*w*/*v*) hydrogel at fixed angular frequency of 1 Hz. The gel was swept from 0.04% to 15% strain and then back to 0.04% strain. This analysis was repeated at least three times for reproducibility assessment.

The mechanical properties of hydrogels stabilized by bolaamphiphile **7** were characterized by rheological measurements. The storage (G′) and loss (G′′) moduli provided information about the visco-elastic properties of gels. The frequency dependence of G′ and G′′ moduli for the hydrogel stabilized by compound **7** (4% *w*/*v*) indicates that G′ exceeded G′′ below a strain of 0.5% and an angular frequency 1 Hz (data not shown). The hydrogel stabilized by **7** exhibits a dominant elastic character (solid-like) as expected for a supramolecular network. The G′ value is 57 kPa at an angular frequency of 1 rad/s for the hydrogel at 4% *w*/*v*. Importantly, compared with the ether analogue (Figure 1a), which possesses a G′ value is 30 kPa in similar rheological conditions, the elastic character of the hydrogel improved with the bolaamphiphile **7**. This behavior is likely due to the presence of carbamate functions in the amphiphile structure. The thixotropic property of the hydrogel **7** was also studied by rheology. The application of a high strain to the sample induces a melting of the gel; this phenomenon was studied by the evolution of the viscoelastic moduli (Figure 3). First, a low strain of 0.04% was applied to hydrogel **7** at 4% (*w*/*v*). In the gel state, G′ was higher than G′′. Then, the hydrogel **7** was suddenly subjected to a higher strain of 15%. At this point, the G′ value was much lower, indicating that the gel liquefies under the stress. With a strain of 0.04% (as in the first step), the sample gradually returns to the gel state and recovers its original strength. The G′ value increases rapidly, and the sample completely recovers to its initial elastic modulus within 30 min.

#### 2.2.3. Gel–Sol Transition Temperature (Tgel)

**Figure 4:**
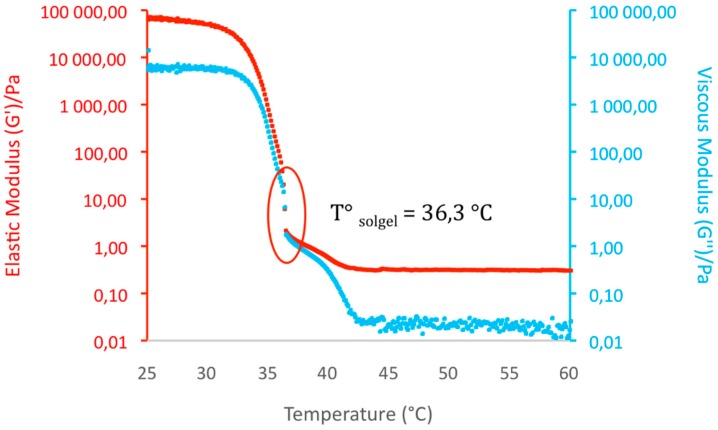
Gel–sol transition temperature determination for bolaamphiphile **7** hydrogel (4% (*w*/*v*)).

The gel–sol transition temperature was determined by rheology (Kinexus^®^ Pro+ rheometer). A temperature ramp from 25 °C to 60 °C (3 °C/min) was used to liquefy the carbamate-based hydrogel. The gel–sol temperature was recorded at the transition between gel and liquid states. An oscillatory stress of 3 Pa and a constant frequency (6.283 rad·s^−1^) were applied during the assay. Graphically, the gel–sol temperature is characterized by the intersection of the two viscoelastic moduli *G*′ and *G*′′. The melting point was observed at 36.3 °C (Figure 4), which is close to 37 °C. This hydrogel could be of interest to the areas for which biomaterials must melt slowly close to 37 °C, such as in the transdermal delivery of drugs.

## 3. Conclusions

In order to expend the current family of glycosyl-nucleoside-lipids, we synthesized a new bolaamphiphile analog featuring carbamate moieties in six steps starting from thymidine. This bioinspired amphiphile, which possesses additional H-bonding capabilities, allows the formation of low-molecular-weight gels (LMWGs) in water at low concentrations (1% *w*/*v*). Importantly, the rheological investigations revealed that the new bolaamphiphile **7** stabilizes hydrogels with higher elastic moduli (*G*′ < 50 kPa) than the diether analogs (*G*′ ≈ 30 kPa) reported previously [15], indicating that the carbamate function contributes favorably to the supramolecular network. As a matter of comparison, dipeptide-based LMWGs previously reported [17] show lower elastic moduli than the bolaamphiphile **7** (*aka* Glycosyl Nucleoside Bola Amphiphile carbamate or GNBA carbamate) hydrogels. The rheological studies also revealed thixotropic properties, emphasizing the possible injectability of the carbamate-based hydrogels.

The molecular approach developed in this report, namely the insertion of hydrogen bond functions in a bolaamphiphile architecture, will be used in the future for the design of new glycosyl-nucleoside lipids for biomedical applications.

## 4. Materials and Methods

### 4.1. General

All commercially reagents and solvents (Alfa-Aesar, Karlsruhe, Germany; Fluka and Sigma-Aldrich, Saint Quentin Fallavier, France) were used without further purification. For reactions requiring anhydrous conditions, dry solvents were used under an inert atmosphere (nitrogen or argon). Analytical thin layer chromatographies (TLC) were performed on pre-coated silica gel F254 plates with a fluorescent indicator (Merck, Fontenay sous Bois, France). The detection of compounds was accomplished with UV light (254 nm). All compounds were characterized using ^1^H and ^13^C nuclear magnetic resonance (NMR) spectroscopy (Bruker Avance DPX-300 spectrometer, Wissembourg, France; ^1^H at 300.13 MHz and ^13^C at 75.46 MHz). Assignments were made by 1H-1H COSY, DEPT, and HSQC experiments. Chemical shifts (δ) are given in parts per million (ppm) relatively to tetramethylsilane (TMS) or residual solvent peaks (CHCl_3_: ^1^H: 7.26 ppm, ^13^C: 77.0 ppm). Coupling constants J are given in Hertz (Hz); peak multiplicity is reported as follows: s = singlet, bs = broad singlet, d = doublet, t = triplet, and m = multiplet. High-resolution mass spectra (HRMS) were performed by the CRMPO (Rennes, France) on a Thermo Fisher Q-Extractive mass spectrometer (Waltham, MA, USA) equipped with an ESI source and recorded in negative mode. 

### 4.2. Synthesis

#### *N*-3-Propargylthymidine (**1**)

Propargyl bromide (80% soln. in toluene, 2.68 g, 18.5 mmol) was added to a mixture of thymidine (3 g, 12.3 mmol), potassium carbonate (2.5 g, 18.5 mmol) and tetra-n-butylammonium iodide (TBAI) (0.45 g, 1.2 mmol) in anhydrous DMF (60 mL). The reaction mixture was stirred for 12 h at room temperature. After concentration to dryness under reduced pressure, water was added to the solid residue, and the product was extracted with ethyl acetate. The organic layer was separated, washed (aqueous 10% KCl then brine), dried (Na_2_SO_4_), and evaporated to give a yellow oil used in the next step without further purification, with a crude yield of 2.82 g (81%).

^1^H NMR (CDCl_3_) δ 1.93 (s, 3H, CH_3_), 2.17 (t, *J* = 2.4 Hz, 1H, CH propargyl), 2.28–2.37 (m, 2H, H-2′), 2.49 (t, *J* = 2.4 Hz, 2H, CH propargyl), 3.81–3.93 (m, 2H, H-5′), 3.98–4.01 (m, 1H, H-4′), 4.52–4.57 (m, 1H, H-3′), 4.68 (d, 2H, CH_2_ propargyl), 6.29 (t, *J* = 6.6 Hz, 1H, H-1′), 7.58 (s, 1H, H-6 thymine).

#### 1-Deoxy-1-azido-2,3,4,6-tetra-*O*-acetyl-β-d-glucopyranose (**2**)

Sodium azide (3.90 g, 60 mmol) was added to a mixture of 2,3,4,6-tetra-*O*-acetyl-β-d-glucopyranosyl bromide [18] (8.22 g, 20 mmol) in anhydrous DMF (100 mL). After stirring for 24 h at 65 °C, the mixture was cooled to room temperature and poured into water (200 mL). The resulting solid was filtered off, washed with water, and dried under vacuum, with a crude yield of 6.92 g (92%). NMR data were identical with those obtained from a commercial sample of the product.

#### *N*-3-[1-((β-d-Glucopyranosidetetraacetate)-1*H*-1,2,3-triazol-4-yl)methyl]thymidine (**3**)

Copper sulfate (0.260 g, 1.63 mmol) and sodium ascorbate (0.645 g, 3.26 mmol) were successively added to a degassed suspension of (**1**) (4.59 g, 16.3 mmol) and 1-deoxy-1-azido-2,3,4,6-tetra-*O*-acetyl glucopyranose (**2**) (6.085 g, 13.3 mmol) in 100 mL of ^t^BuOH/H_2_O (1:1). The mixture was stirred at 75 °C for 7 h. After cooling to room temperature, the solvents were removed under reduced pressure. The resulting solid was washed with water until the washings were colorless. After drying under high vacuum, the resulting white amorphous solid was used in the next step without further purification, with a crude yield of 8.65 g (81%).

^1^H NMR (MeOD) δ 1.94 (s, 3H, CH_3_ thymine), 1.81, 2.00, 2.06 (3s, 12H, OAc), 2.22–2.32 (m, 2H, H-2′), 3.72–3.85 (m, 2H, H-5′), 3.91–0.94 (m, 1H, H-4′), 4.15–4.26 (m, 2H, H-6), 4.30–4.36 (m, 1H, H-3′), 4.39–4.43 (m, 1H, H-5), 5.16–5.30 (m, 3H, H-4 + CH_2_ triazole), 5.52 (dd, *J* = 9.3 Hz, 1H, H-3), 5.60 (dd, *J* = 9.3 Hz, 1H, H-2), 6.1 (d, *J* = 9.0 Hz, 1H, H-1), 6.33 (t, *J* = 6.7 Hz, 1H, H-1′), 7.89 (s, 1H, H-6 thymine), 8.17 (s, 1H, CH triazole).

#### 5′-Deoxy-*N*-3-[1-((β-d-glucopyranosidetetraacetate)-1*H*-1,2,3-triazol-4-yl)methyl] azidothymidine (**4**)

Triphenylphosphine (825 mg, 3.15 mmol), sodium azide (950 mg, 13.1 mmol), and carbon tetrabromide (1.05 g, 3.15 mmol) were added to a solution of (3) (1.5 g, 2.63 mmol) in anhydrous DMF (25 mL). The reaction mixture was stirred at room temperature for 24 h and then treated with 5% aqueous NaHCO_3_ (50 mL). After extracting with dichloromethane, the organic solution was washed with water, followed by drying over Na_2_SO_4_, and concentrated under a reduced pressure. The crude product was purified by column chromatography, eluting with a step gradient of MeOH in CH_2_Cl_2_ (0%–5%) to give the title compound as a white amorphous solid, with a yield of 652 mg (36%).

^1^H NMR (CDCl_3_) δ 1.94 (s, 3H, CH_3_ thymine), 1.83, 2.01, 2.05, 2.08 (4s, 12H, OAc), 2.16–2.25 (m, 1H, H-2′a), 2.36–2.44 (m, 1H, H-2′b), 3.55–3.75 (m, 2H, H-5′), 4.01–4.05 (m, 1H, H-5), 4.06–4.10 (m, 1H, H-4′), 4.14–4.46 (m, 2H, H-6), 4.45 (m, 1H, H-3′), 5.14–5.25 (m, 3H, CH_2_ triazole + H-4), 5.35–5.48 (m, 2H, H-2, 3), 5.84 (d, *J* = 9.0 Hz, 1H, H-1), 6.33 (dd, *J* = 6.7 Hz, 1H, H-1′), 7.37 (s, 1H, H-6 thymine), 7.87 (s, 1H, CH triazole); ^13^C NMR (CDCl_3_) δ 13.45 (CH_3_), 20.31, 20.65, 20.85 (OAc), 36.02 (CH_2_ triazole), 40.48 (C-2′), 52.33 (C-5′), 61.63 (C-6), 67.72 (C-4), 70.26, 72.82 (C-2, 3), 71.51 (C-3′), 75.15 (C-5), 84.49 (C-4′), 85.62, 85,70 (C-1, 1′), 110.57 (C-5 thymine), 122.60 (CH triazole), 134.04 (C-6 thymine), 143.82 (C-4 triazole), 150.68, 163.06 (C=O thymine), 168.97, 169.48, 170.13, 170.76 (C=O acetyl); HRMS *m*/*z* calcd. for C_27_H_34_N_8_O_13_Na 701.21375 [M + Na]^+^, found 701.2138.

#### 5′-Deoxy-*N*-3-[1-((β-d-glucopyranoside)-1*H*-1,2,3-triazol-4-yl)methyl]azido thymidine (**5**)

A stirred suspension of (4) (1.1 g, 1.62 mmol) in anhydrous methanol (20 mL) was treated with a few drops of freshly prepared 1N sodium methoxide solution at room temperature. When TLC (CH_2_Cl_2_/MeOH 8:2) showed complete conversion (approx. 30 min), the reaction mixture was treated with Dowex^®^ 50X2 (Sigma-Aldrich). After filtration, the solvent was removed under reduced pressure. The solid residue was applied to a column of silica gel, and the product eluted with 8:2 CH_2_Cl_2_-MeOH to afford the title compound as a white amorphous solid, with a yield of 0.853 g (77%).

^1^H NMR (MeOD) δ 2.07 (s, 3H, CH_3_), 2.41–2.43 (m, 2H, H-2′), 3.46–3.64 (m, 5H, H-3, 4, 5 + H-5′), 3.68–3.74 (m, 1H, H-6a), 3.85–3.92 (m, 2H, H-2 + H-6b), 4.06+4.13 (m, 1H, H-4′), 4.46–4.51 (m, 1H, H-3′), 5.36 (s, 2H, CH_2_ triazole), 5.60 (d, *J* = 9.2 Hz, 1H, H-1), 6.45 (dd, *J* = 6.7 Hz, 1H, H-1′), 7.73 (s, 1H, H-6 thymine), 8.26 (s, 1H, CH triazole); ^13^C NMR (MeOD) δ 14.12 (CH_3_), 38.00 (CH_2_ triazole), 41.22 (C-2′), 54.16, 71.69, 79.22, 81.92 (C-3, 4, 5, 5′), 63.21 (C-6), 73.21 (C-3′), 74.77 (C-2), 87.21 (C-4′), 88.02 (C-1′), 90.41 (C-1) 112.00 (C-5 thymine), 125.16 (CH triazole), 137.19 (C-6 thymine), 143.19 (C-4 triazole), 152.94, 165.64 (C=O); HRMS *m*/*z* calcd. for C_19_H_26_N_8_O_9_Na 533.17149 [M + Na]^+^, found 533.1716.

#### 1,12-Dodecanediyl-bis-*N*-propargylcarbamate (**6**)

Triethylamine (1.06 g, 10.5 mmol) and propargyl chloroformate (1.18 g, 10 mmol) were added to a cooled (0 °C) suspension of 1,12-diaminododecane (1 g, 5 mmol) in anhydrous dichloromethane (40 mL) over 30 min, and stirring continued for a further 24 h at room temperature. The reaction mixture was poured into water 40 mL), and the organic phase separated. The aqueous phase was extracted with dichloromethane, the organic extracts combined, dried (Na_2_SO_4_), and the solvent removed under reduced pressure. The product was purified by column chromatography eluting with CH_2_Cl_2_/MeOH (99:1 then 98:2) and obtained as a white amorphous solid, with a yield of 0.728 g (40%).

^1^H NMR (CDCl_3_) δ 1.27 (s, 16H, CH_2_), 1.49–1.53 (m, 4H, CH_2_CH_2_N), 2.49 (t, *J* = 2.4 Hz, 2H, CH propargyl), 3.20 (dt, *J* = 6.4, 13.3 Hz, 4H, CH_2_CH_2_N), 4.69 (d, 4H, CH_2_ propargyl) ; ^13^C NMR (CDCl_3_) δ 26.66, 29.20, 29.46 , 29.83 (CH_2_), 41.16 (CH_2_N), 52.30 (CH_2_ propargyl), 74.51 (CH propargyl), 78.38 (Cq propargyl), 155.41 (C=O).

#### 1,12-Diaminododecanediyl-*N*,*N*′-bis-[5′-(4-methyloxycarbonyl-1*H*-1,2,3-triazole-1-yl)-*N*-3-((1-(β-d-glucopyranoside)-1*H*-1,2,3-triazole-4-yl)methyl)-5′-deoxy thymidine] (**7**) (GNBA-carbamate)

Copper sulfate (18.8 mg, 0.118 mmol) and sodium ascorbate (46.8 mg, 0.236 mmol), were successively added to a degassed suspension of (**6**) (0.215 g, 0.59 mmol) and (**5**) (0.546 g, 1.18 mmol) in 20 mL of H_2_O/THF (1:1). The mixture was stirred at 65 °C for 4 h. After cooling to room temperature, the solvents were removed under reduced pressure. The resulting solid was applied to a column of silica gel eluting with CH_2_Cl_2_/MeOH (8:2 to 7:3) to give the title compound as a white amorphous solid, with a yield of 492 mg (65%).

^1^H NMR (MeOD) δ 1.30 (s, 16H, CH_2_), 1.42–1.51 (m, 4H, CH_2_CH_2_NH(CO)), 1.93 (d, *J* = 1.0 Hz, 6H, CH_3_ thymine), 2.31 (dd, *J* = 6.2 Hz, 4H, H-2′), 3.08 (dd, *J* = 6.9 Hz, 4H, CH_2_NH(CO)), 3.36–3.56 (m, 6H, H-3, H-4, H-5), 3.68–3.73 (m, 2H, H-6a), 3.85–3.92 (m, 4H, H-2, H-6b), 4.16–4.21 (m, 2H, H-4′), 4.40–4.46 (m, 2H, H-3′), 4.72–4.76 (m, 4H, H-5′), 5.13 (s, 4H, triazole CH_2_O), 5.52 (s, 4H, triazole CH_2_N), 5.56 (d, *J* = 9.2 Hz, 2H, H-1), 6.21 (dd, *J* = 6.7 Hz, 2H, H-1′), 7.24 (d, 2H, H-6 thymine), 7.97 (s, 2H, H triazole), 8.06 (s, 2H, H triazole); ^13^C NMR (MeOD) δ 11.76 (CH_3_ thymine), 26.39 (CH_2_), 28.95, 29.19, 29.43 (CH_2_ & CH_2_CH_2_O), 35.68 (CH_2_N triazole), 38.13 (C-2′), 40.43 (CH_2_NH(CO)), 51.17 (C-5′), 57.02 (OCH_2_ triazole), 60.96 (C-6), 69.44, 77.00, 79.70 (C-3, C-4, C-5), 71.00 (C-3′), 72.52 (C-2), 84.23 (C-4′), 86.77 (C-1′), 88.18 (C-1), 109.80 (C-5 thymine), 122.84, 125.44 (CH triazole), 135.49 (C-6 thymine), 143.16, 143.37 (C-4 triazole), 150.54, 163.37 (C=O), 157.01 (OC=O); HRMS *m*/*z* calcd. for C_58_H_84_N_18_O_22_Na 1407.58998 [M + Na]^+^, found 1407.5897.

### 4.3. Rheology

All rheological measurements were made using a Kinexus^®^ Pro+rheometer (Malvern Instruments Ltd., Orsay, France). The lower plate was equipped with a Peltier temperature control system and the upper plate was a steel plate–plate geometry (20-mm diameter, gap: 0.3 mm). All experiments were done at 25 °C ± 0.01 °C unless indicated otherwise. To prevent water evaporation and to control the temperature, a solvent trap was used. A sample of bolaamphiphile 7 was placed as a gel between the two plates before assays. All experiments were carried out within the linear viscoelastic regime (LVR). It was determined by an amplitude strain sweep experiment (0.01% to 10% at an angular frequency of 1 Hz or 6.283 rad·s^−1^). Then, a frequency sweep assay (0.6283 to 62.83 rad·s^−1^; applied strain of 0.04% within LVR) allowed the evaluation of elastic (G′) and viscous (G′′) moduli. At least three replicates were analyzed for each sample.

## References

[1] Steed J.W. (2011). Supramolecular gel chemistry: Developments over the last decade. Chem. Commun..

[2] Neelakandan P.P., Hariharan M., Ramaiah D. (2006). A Supramolecular ON–OFF–ON Fluorescence Assay for Selective Recognition of GTP. J. Am. Chem. Soc..

[3] Griffin D.R., Weaver W.M., Scumpia P.O., di Carlo D., Segura T. (2015). Accelerated wound healing by injectable microporous gel. Nat. Mater..

[4] Ziane S., Schlaubitz S., Miraux S., Patwa A., Lalande C., Bilem I., Lepreux S., Rousseau B., le Meins J.-F., Latxague L. (2012). A theromosensitive hydrogel for tissue engineering. eCM.

[5] Tiller J.C. (2003). Increasing the local concentration of drugs by hydrogel formation. Angew. Chem. Int. Ed..

[6] Godeau G., Barthélémy P. (2009). Glycosyl-nucleoside lipids as low-molecular-weight gelators. Langmuir.

[7] Godeau G., Bernard J., Staedel C., Barthélémy P. (2009). Glycosyl-nucleoside-lipid based supramolecular assembly as a nanostructured material with nucleic acid delivery capabilities. Chem. Commun..

[8] Godeau G., Brun C., Arnion H., Staedel C., Barthélémy P. (2010). Glycosyl-nucleoside fluorinated amphiphiles as components of nanostructured hydrogels. Tetrahedron Lett..

[9] Estroff L.A., Hamilton A.D. (2004). Water gelation by small organic molecules. Chem. Rev..

[10] Brard M., Richter W., Benvegnu T., Plusquellec D. (2004). Synthesis and supramolecular assemblies of bipolar archaeal glycolipid analogues containing a cis-1,3-disubstituted cyclopentane ring. J. Am. Chem. Soc..

[11] Meister A., Blume A. (2007). Self-assembly of bipolar amphiphiles. Curr. Opin. Colloid Interface Sci..

[12] Yan Y., Lu T., Huang J. (2009). Recent advances in the mixed systems of bolaamphiphiles and oppositely charged conventional surfactants. J. Colloid Interface Sci..

[13] Meister A., Blume A. (2012). Single-Chain Bolaphospholipids: Temperature-Dependent Self-assembly and Mixing Behavior with Phospholipids. Adv. Plan. Lipid Bilayers.

[14] Blume A., Drescher S., Graf G., Kçhler K., Meister A. (2014). Self-assembly of different single-chain bolaphospholipids and their miscibility with phospholipids or classical amphiphiles. Adv. Colloid Interface Sci..

[15] Latxague L., Ramin M.A., Appavoo A., Berto P., Maisani M., Ehret C., Chassande O., Barthélémy P. (2015). Control of Stem-Cell Behavior by Fine Tuning the Supramolecular Assemblies of Low-Molecular-Weight Gelators. Angew. Chem. Int. Ed. Engl..

[16] Ghosh A.K., Brindisi M. (2015). Organic Carbamates in Drug Design and Medicinal Chemistry. J. Med. Chem..

[17] Raeburn J., Cardoso A.Z., Adams D.J. (2013). The importance of the self-assembly process to control mechanical properties of low molecular weight hydrogels. Chem. Soc. Rev..

[18] Horton D., Lemieux U., Whistler R.L., Wolfrom M.L., BeMiller J.N. (1963). Methods in Carbohydrate Chemistry.

